# Domestic

**Published:** 1866-06

**Authors:** A. Geiger

**Affiliations:** Dayton, Ohio


*Domestic.—Lime Inhalations in Diphtheritic Affections.*—By A. Geiger, M. D., Dayton, Ohio.

I am glad to meet with the *criticisms* of your correspondent, Dr. T. A. Reamy, upon my letter that appeared in the *Reporter* of March 10th, because that by discussion truth is oftentimes elicited, and important facts are thus brought to the notice of the profession, which, otherwise, might remain a long time in oblivion; and because it affords to me an opportunity of stating more specifically the nature of the lime treatment, my method of using it, and the reasons why I was led to adopt it. The letter referred to, was written in a hurried manner, and did not contain a full report of the case, and was not intended for publication, as I stated to you at the time, but simply as further confirmation of the lime treatment, as recommended in the communication that accompanied it, and which is published in the *Reporter* of March 24th. The correction of a small error made by the "compositor" will, I think, lead the doctor to agree with me that I was not mistaken in my diagnosis of the case.

I wrote—"the boy had been exposed on last *Thursday*, (that very cold day which will be remembered by us all,) and at night was taken with hoarse cough, and fever" etc. The compositor has it "last *Saturday*," which makes a difference of two days, and I know that the doctor is not prepared to assume that "spasmodic croup," which he seems to be very sure the case was, would last, accompanied with fever and hoarse cough, and the patient confined to bed for three days, which was really the length of time my patient was sick, before I visited him.

And the doctor will certainly not charge me with recommending the lime inhalation, as a *specific* in diphtheria and membranous croup, when he mentions in the same connection the fact, that I used "hive syrup, calomel, rhubarb," etc. I was aware of the objection, that a comparatively new treatment in a disease so formidable as the one under consideration, would meet in the minds of physicians. And in the same letter stated in language similar to that used by your correspondent: "That I had frequently employed new remedies in the treatment of diseases which had been recommended by others with great confidence, but that in my hands I had often been disappointed in obtaining like favorable results," and that, I feared that others would regard my recommendations with like distrust, and hesitate before adopting the treatment, which was more particularly given in the communication that accompanied my letter. And it was only after having given the treatment what I regarded "a fair trial," that I felt warranted in presenting the matter through your columns to the consideration of the profession at large.

Although I have read the article which appeared in the *Reporter* of Sept. 9th, taken from the *Brit. and For. Med. Chir. Review*, in which favorable report was made of several cases successfully treated by lime inhalations; yet the mode of applying the treatment was so vague, that I could not see in what way the lime was brought in contact with the membrane of the air passages to dissolve it, and did not attempt to use it, although at the time an epidemic of diphtheria was prevailing in this city.

In Wood's *Practice of Medicine* we find it also stated, in speaking of the nature of the false membrane in pseudo membranous croup, that it is dissolved by alkaline solutions, and strong acetic acid, and in the treatment, that alkalies have been administered and were strongly recommended by some writers, supposing that they would have the power to dissolve the secreted membrane, as they are known to do out of the body. That at one time they had obtained quite a reputation, but have since fallen into neglect.

After having obtained the false membrane expectorated by the boy, John Willis, I placed a portion of it in lime water, and found that it dissolved very readily, and then felt satisfied that if some method could be devised to bring the lime in contact with the membrane in the wind-pipe that it would dissolve it there; my ingenuity was put to the task to discover some means by which this desired object would be accomplished.

I obtained some unslacked lime, and placed it in a vessel, and poured upon it first cold water, and upon inhaliag it could discover no effects.

I then poured over another portion *hot* water, and inhaled the steam arising from it. After inhaling it a few moments, I could distinctly feel in the air passages, the smarting action of the lime, and determined to try the effects of the lime inhaled in this way in the next case of diphtheria, or pseudo membranous croup, occurring in my practice.

*In this process, the steam arising from the lime in slacking, contains in it particles of lime which are thus by inhalation brought in contact with the membrane in the windpipe.*

I have used it in this way, and so have others with the results already reported to you, and also at the same time have administered lime water internally; I must confess, rather more *empirical*, than *rational*, but with the hope that it would have some influence in preventing or arresting the formation of the membrane.

Dr. A. Shuleck, of this city, and one of your subscribers, reports to me this morning two cases of pseudo membranous croup successfully treated by the lime inhalations, and it is to be hoped that in its further trial by the profession, it will prove to be a valuable remedy, but it is not claimed as a *specific* in the treatment of this heretofore unmanageable disease.—*Phil. Med. and Surg. Reporter.*

